# The association between self-efficacy and self-management behaviors among Chinese patients with type 2 diabetes

**DOI:** 10.1371/journal.pone.0224869

**Published:** 2019-11-11

**Authors:** Jingjing Yao, Haipeng Wang, Xiao Yin, Jia Yin, Xiaolei Guo, Qiang Sun

**Affiliations:** 1 School of Health Care management, NHC Key Laboratory of Health Economics and Policy Research, Shandong University, Jinan, China; 2 Jinan Central Hospital, Jinan, China; 3 Shandong Center for Disease Control and Prevention, Jinan, China; Chongqing Medical University, CHINA

## Abstract

**Background:**

Self-management is the cornerstone of diabetes care, however, despite the numerous recommendations available for self-management, type-2 diabetes mellitus (T2DM) patients’ performance is suboptimal in China. This study aimed to explore the association between self-efficacy and self-management behaviors among Chinese T2DM patients, which might provide evidence to inform effective self-management interventions for these patients.

**Methods:**

A cross-sectional survey was conducted using a multi-stage stratified randomized sampling in Shandong Province, China. The Diabetes Empowerment Scale-Short Form (DES-SF) was used to measure patients’ self-efficacy to manage diabetes. Latent class analysis (LCA) was used to explore the observed classes of self-management behaviors (dietary control, physical exercise, regular medication and self-monitoring of blood glucose). A two-class solution for self-management behaviors was tested to be the fittest based on LCA; we labelled active and inactive self-management groups. Univariate and multivariate logistic regression analysis were used to examine the associations between self-efficacy and self-management behaviors.

**Results:**

A total of 2166 T2DM patients were included in the analysis. The mean DES-SF score was 31.9 (standard deviation: 5.2). The estimated proportions of T2DM in the active and inactive groups were 54.8% and 45.2%, respectively. The multivariate logistic regression showed that higher DES-SF score was significantly associated with higher possibility of active self-management behaviors (odds ratio = 1.06; 95% confidence interval: 1.04–1.08).

**Conclusions:**

Self-efficacy in managing diabetes is associated with self-management behaviors among Chinese T2DM patients. To improve self-management behaviors, multiple strategies should be conducted to improve patients’ self-efficacy.

## Introduction

Type 2 diabetes mellitus (T2DM) has rapidly become one of the most common non-communicable diseases (NCDs) globally, and one of the most challenging public health issues [1]. The International Diabetes Federation (IDF) estimated that 425 million people had diabetes worldwide in 2017, and this will rise to 627 million by 2045 [2]. In China, a rapid increase in the prevalence of diabetes has been reported. Comparison of the latest national diabetes survey in 2013 with the first in 1981 shows diabetes prevalence has increased 17-fold [3–4]. The estimated prevalence by the World Health Organization (WHO) in 2016 showed that China had about 104 million diabetes patients in adults, of which, T2DM accounts for about 95% of the cases [5].

T2DM, as a chronic disease, affects people throughout their lifetime. T2DM patients are tasked with performing effective self-management. According to the Global Guideline for Type 2 Diabetes, four basic diabetes self-management skills were recommended for T2DM patients, which included dietary control, physical exercise, regular medication and self-monitoring of blood glucose [6]. Multiple clinical trials and reviews have confirmed the vital role of self-management in reducing blood glucose and improving quality of life among DM patients [7–9]. However, patients’ adherence to self-management is far from satisfactory worldwide [10–12].

In the literature, many factors have been reported affecting self-management behaviors among T2DM patients; these include socioeconomic positions, diabetes knowledge, heath beliefs, attitudes, motivation and social support, etc [13–18]. One of the key factors is self-efficacy. The concept of self-efficacy is based on social cognitive theory [19], reflecting individuals’ belief in their capability to perform specific behaviors necessary to achieve their goals. Improvements in patients’ self-efficacy have been used as a mechanism to improve health-related behaviors among those with chronic diseases, such as hypertension, DM, chronic obstructive pulmonary disease, arthritis and cardiovascular diseases [20]. Many studies have also shown the direct influence of self-efficacy on physical and mental health [21].

In China, self-efficacy has been shown to be positively associated with medication adherence and walking exercise among hypertension patients [22–23]. Further studies have found self-efficacy was an important predictor of diabetes patients’ adherence to self-monitoring of blood glucose and dietary control [24–25]. However, no study has identified the relationship between self-efficacy and the cluster of self-management behaviors recommended for diabetes management. This study therefore aimed to examine the association between self-efficacy and self-management behaviors among Chinese T2DM patients, which might provide evidence to inform effective self-management interventions for them in China.

## Materials and methods

### Study design and patient selection

This was a cross-sectional study in Shandong Province, China. Shandong contains 17 prefectures and 140 counties (districts), and had a total population of nearly 99 million in 2016. The estimated number of T2DM patients was about 980,000 (prevalence: 9.3%) [26]. Up to 2016, nearly 30% the total T2DM patients were registered in the NCDs management system.

This study selected respondents from those patients registered in the NCDs management system. The sample size was calculated using Krejcie and Morgan’s formula [27]. Based on this calculation, the sample size was estimated as 2066 (at 95% confidence and 5% margin of error). A larger sample size of 2520 patients were targeted to cater for a 10% attrition. A multi-stage stratified randomized sampling was employed as follows. First, four representative prefectures (Qingdao, Weifang, Jinan and Heze) were selected based on their geographic location and economic development status within the province. Then, three subdistricts (urban) and three towns (rural) were randomly selected from each of the four prefectures. Furthermore, three communities from each subdistrict and three villages from each town were randomly selected. In total, 36 urban communities and 36 rural villages were selected. Finally, 35 T2DM patients were randomly recruited from each selected community and village. The inclusive criteria of the study were: (1) diagnosis of T2MD diabetes based on WHO criteria for one year, (2) age 35 to <80 years and (3) ability to normally communicate with others.

### Ethics statement

This study was conducted in accordance with the Declaration of Helsinki. The protocol was approved by the ethics committee of School of Health Care Management, Shandong University, China. All participants were informed prior to investigation, and consent forms were signed by participants themselves. For these who can’t sign their names, the ethics committee approved that consent forms can be signed by their relatives or data collectors at the request of the participants.

### Data collection

The study was conducted between August and October in 2017. Patient information was collected using a structured questionnaire. Trained data collectors from Shandong University delivered the questionnaire by face-to-face interview. To ensure the questionnaire understood by the patients appropriately, all interviewers were required to make uniform explanations to the interviewees based on their local culture and customs. To ensure the quality, the dedicated supervisors carefully checked all completed questionnaires each day after interviews. Briefly, the questionnaire inquired on: (1) the patient’s basic information and health status, such as residence, gender, age, marital status, education level, household income, duration of diabetes diagnosis and diabetes comorbidity; (2) self-efficacy to manage diabetes and (3) self-management behaviors, including dietary control, physical exercise, regular medication and self-monitoring of blood glucose.

### Assessed variables

In this study, the outcome variable was patients’ self-management behaviors with four dimensions, including dietary control, physical exercise, regular medication, and self-monitoring of blood glucose. Self-developed questions were used to measure the four behaviors based on expert consensus and previous works [28, 29]. In accordance with recommendations from the *China guidelines for type 2 diabetes* [30], all dimensions of self-management behaviors were treated as binary indicators. Dietary control was measured using three questions: “Do you intentionally eat less food with high carbohydrate everyday?”; “Do you intentionally eat less food with high fat everyday?” and “Do you intentionally not intake much caloric everyday?”. Patients responding “yes” for all these questions were identified as dietary control. The questions for physical exercise were “What type of physical excise did you often take (low-intensity exercise, middle-intensity excise or high-intensity excise)?”, “On average, how often did you do your exercise in the last month?” and “On average, how long did you do your exercise each time in the last month?”. Patients who reported doing middle-intensity exercise ≥150 minutes per week were regarded as physical exercise. Regular medication was measured by asking “Over the past 2 weeks, were there any days when you did not take your diabetes medicine?”; “When you feel like your diabetes is under control, do you sometimes stop or cut back taking your medicine?”; and “Have you ever cut back or stopped taking your medication without telling your doctor because you felt worse when you took it?”. These who answered “no” to all these questions were regarded as regular medication. The question for the self-monitoring of blood glucose was “How often did you monitor your blood glucose by yourself or your family members?” Patients who reported monitoring their blood glucose at least every month were regarded as having self-monitoring behavior.

The exposure variable was patients’ self-efficacy to manage diabetes. Patients’ self-efficacy was measured by the Diabetes Empowerment Scale-Short Form (DES-SF) with eight items [31]. The Cronbach's alpha coefficient of internal consistency reliability of DES-SF was 0.85 among Chinese T2DM patients [32]. Responses for each item was rated on a five-point Likert scale, where 1 = totally disagree, 2 = somewhat disagree, 3 = neither agree nor disagree, 4 = somewhat agree, and 5 = totally agree. Total scores ranged from 8 to 40, with a higher score indicating a higher self-efficacy level.

Covariates included demographics and clinical variables. Demographics included residence (urban or rural), gender (male or female), age (35–49, 50–64 or 65–79 years), marital status (single or married, with single encompassing unmarried, divorced or widowed), education level (no formal education, primary school, or junior school or higher), household income level (Q1:<422, Q2:423–870, Q3:871–1739 and Q4: 1740–8122 US$, classified by the quartile of the household income per capita). Clinical variables included duration of diagnosis (<5, 6–10, >10 years) and diabetes comorbidity (no or yes). Comorbidity meant the presence of any diabetes-related diseases or conditions (i.e., diabetic nephropathy, diabetic eye complications, diabetic foot, diabetic cardiovascular complications, diabetic cerebrovascular disease, or diabetic neuropathy).

### Statistical analysis

Descriptive statistics (mean [M] and standard deviation [SD]) was presented for numerical variables. Categorical variables such as residence, gender, age groups and duration of disease groups were presented as their frequency and percentage. Latent class analysis (LCA) was used to explore the observed classes of self-management behaviors [33]. We began with a baseline one-class model and proceeded to test models with successively larger numbers of classes. The choice of the optimal number of classes was based on the comparison of the various class-size models using the following criteria: (1) the Bayesian Information Criteria (BIC), where smaller BIC value indicates better fit model; (2) Akaike Information Criteria (AIC), where smaller AIC value indicates better fit model. Lin and Dayton [34] indicated that BIC tended to be superior to AIC in terms of accuracy for a bigger samples size (n>1000). Further, we also assessed the entropy of each model as another indicator for class separation, and performed Lo-Mendell-Rubin Likelihood Ratio tests (LMRLR) to test the significance of the difference in the likelihoods of two models [35]. Univariate and multivariate logistic regression analyses were used to examine the associations between self-efficacy and self-management behaviors, and to identify other factors associated with self-management behaviors. The stepwise backward likelihood ratio method with a p-value of <0.05 was used in model selection. Data were analyzed using Stata 15 (StataCorp LLC, College Station, TX, USA) for Windows.

## Results

### Participant characteristics

As shown in Table 1, a total of 2166 T2DM patients were included in the analysis, comprising 1070 patients from urban areas and 1096 from rural areas. The mean age of the participants was 63.5 years, and 49.9% were≥65 years old. Majority of the participants were women (65.4%) and the married (86.1%). With respect to education level, 32.8% had no formal education, 33.1% had primary school education, and 34.1% of the participants had junior school or higher education. The median annual household income per capita was 6,000 Yuan (about US$869). Overall, 38.5% had been diagnosed with diabetes within the preceding 5 years, and 35.7% patients reported having a diabetes comorbidity.

**Table 1:** Basic characteristics of the participants (N = 2166).

Characteristics	N	Percent (%)
Residence		
Urban	1070	49.4
Rural	1096	50.6
Gender		
Male	749	34.6
Female	1417	65.4
Age in groups, years		
35~49	121	5.6
50~65	965	44.6
65~79	1080	49.9
Marital status		
Single	301	13.9
Married	1865	86.1
Education level		
No formal education	711	32.8
Primary school	716	33.1
Junior school and higher	739	34.1
Household income per capita, USD dollars		
≤422	542	25.0
423–870	541	25.0
871–1739	541	25.0
≥1740	542	25.0
Duration of diabetes, years		
<5	833	38.5
6~10	680	31.4
>10	653	30.2
Diabetes comorbidity		
No	1393	64.3
Yes	773	35.7

### Patients’ self-efficacy and self-management behaviors

As shown in Table 2, the mean DES-SF score was 31.9 (SD = 5.2). The score of each item ranged from 3.9 to 4.1. The highest-scored item was “…ask for support for having and caring for my diabetes when I need it” (M = 4.1, SD = 0.8) and the lowest was “…know enough about myself as a person to make diabetes care choices that are right for me” (M = 4.1, SD = 0.8). The proportions of patients performing well self-management in medication adherence, dietary control, physical exercise and self-monitoring of blood glucose were 75.8%, 74.5%, 61.0%and 25.8%, respectively.

**Table 2:** Self-efficacy and self-management behaviors of study patients (N = 2166).

Items	
**Diabetes Empowerment Scale-Short Form, Mean (standard deviation)**	
I am confident that I am able to:	
…know what part(s) of taking care of my diabetes that I am dissatisfied with.	3.90 (1.02)
…turn my diabetes goals into a workable plan.	3.99 (0.94)
…try out different ways of overcoming barriers to my diabetes goals.	3.25 (0.92)
…find ways to feel better about having diabetes.	4.07 (0.92)
…know the positive ways I cope with diabetes-related stress.	4.01 (0.92)
…ask for support for having and caring for my diabetes when I need it.	4.12 (0.83)
…know what helps me stay motivated to care for my diabetes.	4.02 (0.87)
…know enough about myself as a person to make diabetes care choices that are right for me.	3.87 (1.06)
Total:	31.93 (5.17)
**Diabetes self-management behaviors, N (%)**	
Dietary control	1613 (74.47)
Regular medication	1415 (75.83)
Physical exercise	1322 (61.03)
Self-management of blood glucose	559 (25.81)

### Latent class analysis of self-management behaviors

As shown in Table 3, models with one to four latent classes were estimated, where the one-class model was deemed a baseline. Models were then compared based on the LCA criteria outlined in the analyses section. After careful review of all the models, the two-class solution for self-management behavior best satisfied the selection criteria: (1) Bayesian information criterion was at the minimum; (2) Akaike information criterion was relatively small; (3) LMRLR indicated two- class model was the best-fitting model(LMRLR compares n versus n-1 class models and reject the null hypothesis that n-1 class fits the data better than n class model, if p<0.05).

**Table 3:** Model fit statistics of the one- to four-class latent class analysis models.

# of classes	Log likelihood	Degree of freedom	BIC	AIC	LMRLR testing the null hypothesis	P-value for LMRLR
1	-4904.4	513	10657.6	10634.9	--	--
**2**	**-4869.1**	**412**	**10629.7**	**10578.6**	**Class 1 vs Class 2**	**0.03**
3	-4862.7	401	10657.7	10578.2	Class 2 vs Class 3	0.30
4	-4862.0	390	10672.0	10581.1	Class 3 vs Class 4	0.08

**Note**: Bold text signifies the selected model.

**Abbreviations**: BIC: Bayesian information criterion; AIC, Akaike information criterion; LMRLR, Lo-Mendell-Rubin Likelihood Ratio tests.

**Fig 1:**
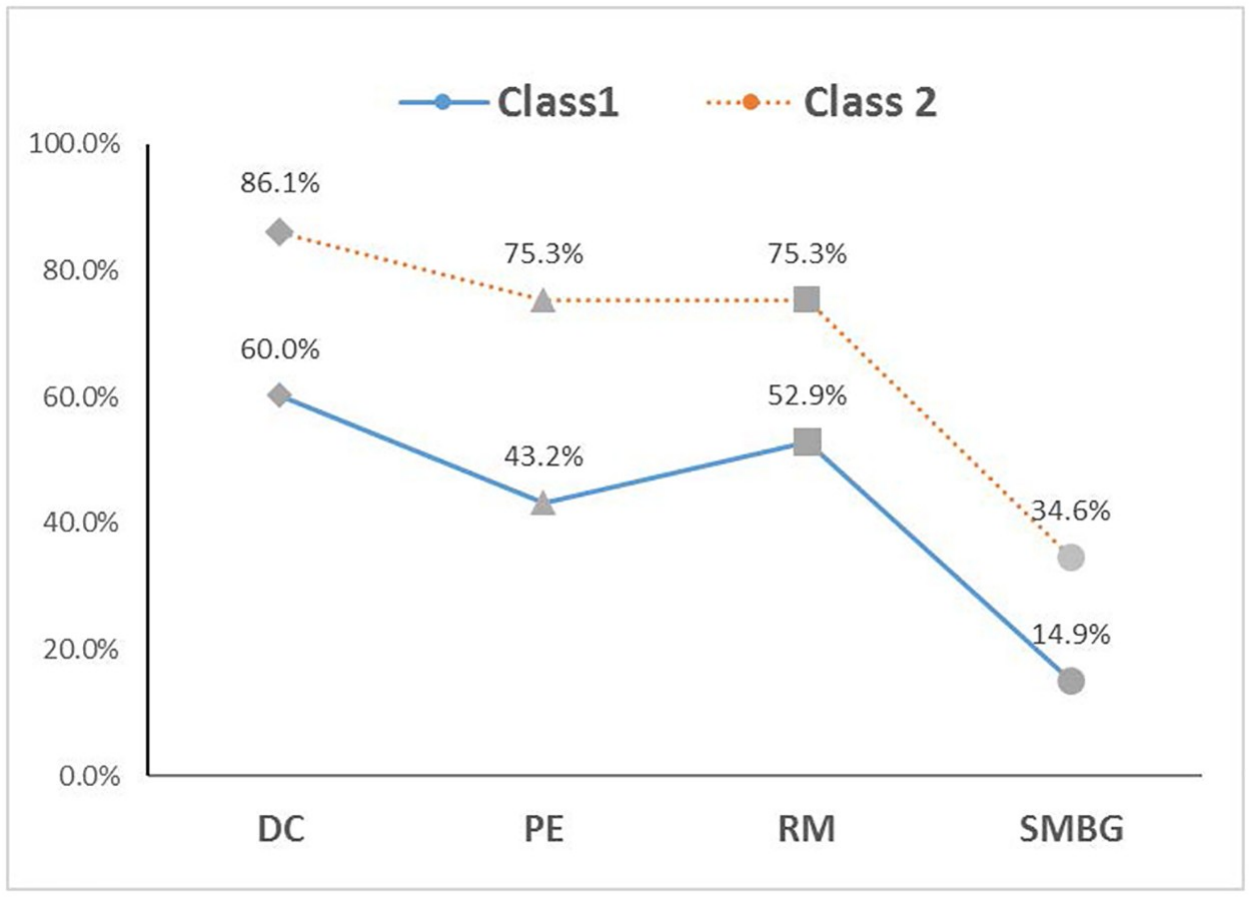
Item-response probabilities of self-management behaviors for the two-class model: Probability of endorsing an item given a latent class. **Abbreviations**: DC, Dietary Control; PE, Physical Exercise; RM, Regular Medication; SMBG, Self-Monitoring of Blood Glucose.

Fig 1 presented the estimated item-response probabilities for self-management behaviors in each of the two latent classes. It showed that class 2 had relatively higher probabilities of self-management than class 1 in all aspects of self-management behaviors, including dietary control (86.1% vs. 60.0%), physical exercise (75.3% vs. 43.2%), regular medication (75.3% vs. 52.9%) and self-monitoring of blood glucose (34.6% vs. 14.9%). We named class 1 and class 2 the inactive and active self-management groups. The estimated proportions of the two classes were 45.2% and 54.8%, respectively.

### The relationship between self-efficacy and self-management behaviors

As shown in Table 4, higher DES-SF score was significantly associated with higher possibility of active self-management behaviors. In univariate results, the patients’ possibility of such behaviors increased 1.25 (95% Confidence Interval [CI]: 1.16–1.35) times for every one-point increase in DES-SF score. In multivariate results, after adjusting for covariates, this possibility decreased in magnitude but remained positively significant (Odds Ratio [OR] = 1.06; 95%CI: 1.04–1.08). Other factors significantly associated with active self-management behaviors were residence, education level, household income per capita, duration of diabetes and diabetes comorbidity. Participants who had a higher education level (P<0.001), higher household income per capita (P<0.05), longer duration of diabetes (P<0.001) and a diabetes comorbidity (OR = 1.26; 95%CI: 1.04–1.52) were more likely to have active self-management behaviors than their counterparts. Those who lived in rural areas were less likely (OR = 0.73; 95%CI: 0.61–0.88) to have active self-management behaviors than those in urban areas.

**Table 4:** Logistic regression analysis of factors affecting patients’ self-management behavior (N = 2166).

Characteristics	Univariate analysis		Multivariate analysis	
	OR (95%CI)	*P*	OR (95%CI)	*P*
Perceived self-efficacy score	1.25 (1.16~1.35)	0.00	1.06 (1.04–1.08)	0.00*
Residence: (Reference: Urban)				
Rural	0.67 (0.57~0.80)	0.00	0.73 (0.61–0.88)	0.01*
Gender (Reference: Male)				
Female	0.95 (0.80~1.14)	0.57	0.99 (0.82–1.19)	0.92
Age in groups, years (Reference: 35~49)				
50–64	1.15 (0.79~1.68)	0.47	1.08 (0.73–1.60)	0.70
65–79	1.10 (0.76~1.61)	0.61	1.07 (0.72–1.58)	0.74
Marital status (Reference: Single)				
Married	1.23 (0.96~1.56)	0.10	1.16 (0.90–1.51)	0.25
Education level (Reference: No formal education)				
Primary school	1.21 (0.98~1.49)	0.08	1.19 (0.92~1.43)	0.25
Junior school and higher	1.62 (1.32~2.00)	0.00	1.52 (1.18~1.95)	0.00*
Household income per capita, USD dollars (Reference: ≤422)				
423–870	1.27 (1.00~1.62)	0.06	1.19 (0.93–1.52)	0.17
871–1739	1.22 (0.96~1.54)	0.11	1.09 (0.85–1.40)	0.50
≥1740	1.69 (1.33~2.15)	0.00	1.43 (1.11–1.85)	0.01*
Duration of diabetes, years (Reference: <5)				
6~10	1.15 (0.94~1.41)	0.18	1.11 (0.90–1.37)	0.31
>10	1.52 (1.23~1.87)	0.01	1.46 (1.17–1.82)	0.02*
Diabetes comorbidity (Reference: No)				
Yes	1.26 (1.05~1.50)	0.01	1.26 (1.04–1.52)	0.02*

*Significantly associated with self-management behaviors at p<0.05.

**Abbreviations**: OR, Odds Ratio; CI, Confidence Interval.

## Discussion

Our study applied LCA to classify self-management behaviors among Chinese T2DM patients into active and inactive self-management groups. The former’s probability of performing self-management was greater than that of the latter in each diabetes self-management behavior item. Based on self-management behaviors classification, this study further explored the relationship between self-efficacy and self-management behaviors. The result revealed that patients’ self-efficacy to manage diabetes was significantly associated with self-management behaviors among Chinese T2DM patients, after controlling for other demographics as well as health-related factors.

Our study found that the performance of self-management among T2DM patients was poor, with only 54.8% patients in active self-management groups. This result was similar with a study in Chongqing province, China, which showed that only a half of DM patients have good self-management behaviors [12]. Meanwhile, our study revealed that DM patients had worst self-management performance in self-monitoring of blood glucose when compared with other dimensions of self-management behaviors. This discrepancy can be partly explained by patients’ financial barrier and the uneven provision of diabetes management services in Chinese primary health care. From the perspective of patients’ financial barrier, 95% Chinese population were covered by social basic health insurance scheme, which includes the rural new cooperative medical scheme, urban resident-based basic medical insurance scheme, and urban employee-based basic medical insurance scheme [36]. However, all these insurance schemes did not pay for the blood glucose monitoring equipment and the test strips, which may bring a heavy financial burden for the patients. From the perspective of delivery of primary care services, DM management program, as one of the key contents of the national essential public health services (EPHS), was implemented since 2009 when the new round of health system reform was initiated. Patients registered in EPHS can freely receive lifestyle and medication guidance from their contracted primary health workers [37]. However, self-monitoring of blood glucose guidance was hardly available for these patients. In addition, fear of needles may also be a barrier of self-monitoring of blood glucose among T2DM patients [38]. Our results hinted that more educational and financial support should be given to these patients to improve their self-monitoring blood glucose.

Self-efficacy was a crucial psychological factor, which reflects patients’ confidence in managing their disease. Consistent with previous studies, in which self-efficacy was found as a strong predictor of self-management behaviors among patients with hypertension, arthritis and cardiovascular diseases [20,39,40], this study showed that self-efficacy was positively related to self-management behaviors among Chinese T2DM patients. Some possible explanations for that were that patients with higher self-efficacy were more likely to acquire disease-related knowledge and seek help from the doctors and their family members, which was greatly useful for them to maintain the self-management behaviors. Moreover, previous studies confirmed that self-efficacy was associated with better quality of life, less depression and lower HbA1c in diabetes patients [41]. Therefore, it is necessary to promote the self-efficacy intervention in clinical setting. On the one hand, physician or health educators could use existing measures (e.g., DES-SF) to evaluate patients’ self-efficacy in 3–5 minutes, and to identify their barriers to perform self-management behaviors during outpatient visits. On the other hands, some technical methods can be used by the physicians to inspire patients’ confidence in managing their disease, such as motivational interviews, shared planning and peer leaders [42–44]. In addition, some innovative delivery ways can be introduced to the self-efficacy intervention, such as messaging interventions and family-based intervention [45,46], which may facilitate the exchange of information between the patients and physicians and help the patient get more support from family members or physicians.

Our study found that rural patients had worse performance in self-management than the urban ones. This disparity may be related to the different socioeconomic development levels between urban and rural areas. Patients in urban areas usually had higher incomes and education levels, which had been found positively associated with self-management behaviors. Furthermore, this disparity may be due to urban-rural inequity in quality of healthcare services. In China, primary health care for diabetes in urban areas was mainly provided by general practitioners (GPs), but by village doctors in rural areas. Compared with village doctors, GPs usually have more comprehensive medical knowledge and skills, which may positively influence the quality of diabetes care. Our study suggested that more measures should be taken to eliminate the difference in quality of primary care between and urban and rural areas.

Consistent with Sarkar [47], our study showed that longer duration of diabetes and having a diabetes comorbidity were associated with better self-management behaviors. Some common explanations for this were that patients with longer duration of diabetes may be more susceptible to diabetes complications, more dependent on self-management and may have longer time to develop a habit of self-management behaviors [10]. Our result suggested that self-management education and support for patients with diabetes should to be an ongoing process, starting at the time of diagnosis.

Several limitations of the study should be noted. First, due to the cross-sectional nature of the study, inferences about causality between self-efficacy and self-management behaviors could not be made. Secondly, self-management behaviors were measured by self-developed questionnaire. Misclassification for them might occurred for lack of established criteria despite strict quality control during the interviews.

In conclusion, self-efficacy is a strong predictor of self-management behaviors among T2DM patients. The interventions that contribute to improving patients’ self-efficacy should be piloted in routine practice, especially in rural clinics, focusing on the patients with lower economic status and the newly diagnosed.

## Supporting information

S1 File The questionnaire used in the study (Chinese version). (PDF)

S2 File The questionnaire used in the study (English version). (PDF)

S3 File Ethics committee approve form. (PDF)
