# Supplementary material for: The association between self-efficacy and self-management behaviors among Chinese patients with type 2 diabetes
Source: PLoS One. 2019 Nov 11;14(11):e0224869. doi: 10.1371/journal.pone.0224869 (PMC6844544; doi:10.1371/journal.pone.0224869)
Supplement: S1 File — (PDF) [file pone.0224869.s001.pdf]

居民健康问卷

调查对象姓名：______________　　个人编码：__________________

家庭地址：_____________ 县_________乡(镇)__________________ 村

调查员：______________　　　　问卷审核员：____________

知情同意书

我们非常真挚地邀请您参加一个关于糖尿病自我行为的调查。该课题是由山东大学医药卫生管理学院的孙强教授主持，主要目的是了解山东省 2 型糖尿病患者的自我管理行为状况。该调查主要采用面对面询问的方式，调查的时间大概为 10-20 分钟。

该调查的数据主要用于科学研究，无任何商业利益。课题组所有成员均将严格执行信息保密规定，确保您个人信息的安全。除必要情况下，伦理委员会和政府管理部分进行数据审查，在未经您容许的情况下，数据禁止外泄他人。

本次调查，纯凭个人意愿。如果有任何疑问您可以向我们的研究人员进行咨询；同时，在参与的过程中，您也可以随时选择退出。且不会对您产生任何健康或者利益损失。

知情同意书签名：

本人已了解这次调查，并对调查员的说明感到满意，我自愿参加这项询问调查及提供相关信息。

调查对象签名_________

签名日期：　　年　　月　　日

第一部分　个人基本情况

问题	答案
**A1.** 居住地:（1） 城市;(2） 农村	
**A2.** 性别：（1)男；(2)女	
**A3.** 您的年龄为:________岁	
**A4.** 您的婚姻状况：（1)未婚；(2)已婚；(3)离异或分居；(4)丧偶	
**A5.** 您的文化程度：<br>(1)没上过学；(2)小学；(3)初中；(4)高中/技校/中专/中技；(5)大专及以上	
**A6.** 过去一年，您的居住方式：<br>(1)配偶;(2)独居;(3)子女;(4)配偶及子女;(5)弟兄姐妹;(6)其他	
**A7.** 过去一年，您家的常住人口数:________人	
**A8.** 过去一年，您家的总收入:________(万元)<br>提示：收入来源包括，种植收入、打工收入、资产收入和其他收入	
**A9.** 您诊断为糖尿病的时间为：________年	
**A10.** 您是否有糖尿病的并发症**(多选)**：<br>(1)糖尿病眼病；<br>(2)糖尿病足病；<br>(3)糖尿病心血管病；<br>(4)糖尿病脑血管病<br>(5)糖尿病肾病；<br>(6)其他，注明__________<br>(7） 没有	

第二部分　糖尿病患者管理状况

问题	答案
**B1.** 您平时是否会控制每日饮食总量？ (1)是；(2)否	
**B2.** 您平时是否会避免吃高脂肪的饮食(如：油炸食品、糕点、肥肉)？(1)是；(2)否	
**B3.** 您平时是否会避免食用含糖量高的食物(如：糖、某些水果比如西瓜、蛋糕等)？ (1)是；(2)否	
**B4.** 经常参加的体育锻炼是？(1)低强度（走路、打太极拳类等)；(2)中等强度（快走、跳舞)；(3)高强度（跑步、骑车、游泳、打球）	
**B5.** 平均每周参加几次这种身体活动？________次	
**B6.** 您平均每次参加身体活动的时间为？(1)小于 15 分钟；(2)15-30 分钟；（3）30-60 分钟 （4）60 分钟以上	
**B7.** 过去 2 周内，是否有一天或者几天您忘记服药？ (1)是；(2)否	
**B8.** 过去半年内，当您觉得症状加重时，您是否未告知医生而自行减少药量或者停止服药？ (1)是；(2)否	
**B9.** 过去半年内，当你觉得自己的病情得到控制时，您是否停止过服药？(1)是；(2)否	
**B10.** 您进行血糖自我测试的频率为？ ___________	

第三部分　自我管理效能

问题	答案
**C1.** 我相信：我知道日常的糖尿病管理中哪些方面做的不到位<br>(1)非常认同；(2)认同；(3)一般；(4)不认同；(5)非常不认同	
**C2.** 我相信：我可以通过一个可行的管理方案实现我的糖尿病控制目标<br>(1)非常认同；(2)认同；(3)一般；(4)不认同；(5)非常不认同	
**C3.** 我相信：我可以想出一些办法去克服障碍去实现目标<br>(1)非常认同；(2)认同；(3)一般；(4)不认同；(5)非常不认同	
**C4.** 我相信：我可以让自己心理上更加适应糖尿病<br>(1)非常认同；(2)认同；(3)一般；(4)不认同；(5)非常不认同	
**C5.** 我相信：我知道运用积极的方式应对糖尿病带来的心理压力<br>(1)非常认同；(2)认同；(3)一般；(4)不认同；(5)非常不认同	
**C6.** 我相信：我当自己需要护理和治疗方面的帮助或者支持时可以有效进行求助<br>(1)非常认同；(2)认同；(3)一般；(4)不认同；(5)非常不认同	
**C7.** 我相信：我知道如何鼓励自己进行糖尿病的管理<br>(1)非常认同；(2)认同；(3)一般；(4)不认同；(5)非常不认同	
**C8.** 我相信：我对自己的病情有足够的了解，知道哪些医疗服务是对自己有帮助的 (1)非常认同；(2)认同；(3)一般；(4)不认同；(5)非常不认同	
