# Supplementary material for: The association between self-efficacy and self-management behaviors among Chinese patients with type 2 diabetes
Source: PLoS One. 2019 Nov 11;14(11):e0224869. doi: 10.1371/journal.pone.0224869 (PMC6844544; doi:10.1371/journal.pone.0224869)
Supplement: S2 File — (PDF) [file pone.0224869.s002.pdf]

Questionnaire for type-2 diabetes patients

Name of interviewee：____________ Code of interviewee：_______________

Address ： ______County (District) _____Town (Subdistrict)_____Village (community)

Name of Interviewer: ________________

Name of Supervisor: _________________

Informed Consent Form

You will be invited to participate in a diabetic self-management behaviors study. This informed consent form provides you with some information. Please read the following information and decide whether to participate in this study.

This study was design by Prof. Sun in Shandong university. The main purpose of this study is to understand the diabetic patients' self-management behaviors in Shandong province, China. The survey was conducted via face-to-face interviews, which will last about 10-20 minutes.

The information obtained in this survey is only for scientific research without any commercial interests. All our team members are required to keep your personal information confidential and not disclose it to anyone outside the study group without your permission. When necessary, only ethics committee members and government authorities can access your research materials.

Whether or not to participate in the research is entirely up to you. If you have any questions about this research, you can ask the researcher. You can also refuse to participate in the study or withdraw from the study at any time during the study, which will not affect your relationship with the researcher and will not affect your rights.

If you agree to this survey, please sign your name below (If you can't sign, you can assign one of your family members or our investigator to sign it).

Signature:
Data:

Part A: Demographics and diseases characteristic

Questions:	Answer
**A1.** Residence： (1) Urban ;(2) Rural	
**A2.** Gender： (1)Male；(2) Female	
**A3.** Age： ________ years old	
**A4.** Marital status： (1) unmarried； (2) married； (3) divorced \separated\ widowed	
**A5.** Educational level：<br>(1) No formal education；(2) Primary school；(3) Junior high school ；(4) Senior high school and higher； (5) College and university education	
**A6.** Family members: _____________	
**A7.** The total income of your family in the past year?<br>Hint: family income may from agricultural products, salary, asset interest	
**A8.** When you were diagnosed with diabetes?	
**A9.** Do you have the following diabetes-related comorbidities, **You can choose more than one answer(s)**<br>(1) diabetic nephropathy<br>(2) diabetic eye complications<br>(3) diabetic foot<br>(4) diabetic cardiovascular complications<br>(5) diabetic cerebrovascular disease<br>(6) diabetic neuropathy<br>(7) others, which was/were________<br>(8) none	

Part B: Self-management Behaviors

Questions:	Answer
**B1.** Do you intentionally eat less food with high carbohydrate everyday? (1) Yes; (2) No	
**B2.** Do you intentionally eat less food with high fat everyday? (1) Yes; (2) No	
**B3.** Do you intentionally not intake much caloric everyday? (1) Yes; (2) No	
**For BI-B3, patients responding "yes" for all questions were identified as dietary control.**	
**B4.** What type of physical excise did you often take?" (1). low-intensity, e.g. walking, doing Tai Ji; (2). middle-intensity, e.g. jogging and dancing; (3). high-intensity excise e.g. running and swimming	

**B5.** On average, how often did you do your exercise in the last month?	
**B6.** On average, how long did you do your exercise each time in the last month?” ___hours	
**For B4-B6, patients who reported doing middle-intensity exercise ≥150 minutes per week were regarded as having good physical exercise.**	
**B7.** Over the past 2 weeks, were there any days you did not take your diabetes medicine	
**B8.** Have you ever cut back or stopped taking your medication without telling your doctor because you felt worse when you took it	
**B9.** When you feel like your diabetes is under control, do you sometimes stop taking your medicine	
**For B7-B9, patients who positively answered all questions were regarded as having regular medication**	
**B10.** How often did you monitor your blood glucose by yourself or your family members	
**For B10, Patients who reported monitoring their blood glucose at least one time per month were regarded as having good self-monitoring.**	

Part C: Diabetes Empowerment Scale-Short Form

**Questions:**	totally disagree,	somewhat disagree	neither agree nor disagree	somewhat agree	totally agree
I am confident that I am able to:					
**C1.** …know what part(s) of taking care of my diabetes that I am dissatisfied with.					
**C2.** …turn my diabetes goals into a workable plan.					
**C3.** ...try out different ways of overcoming barriers to my diabetes goals.					
**C4.** ...find ways to feel better about having diabetes.					
**C5.** ...know the positive ways I cope with diabetes-related stress.					
**C6.** ...ask for support for having and caring for my diabetes when I need it.					
**C7.** ...know what helps me stay motivated to care for my diabetes.					
**C8.** ...know enough about myself as a person to make diabetes care choices that are right for me.					
