# Supplementary material for: The association between self-efficacy and self-management behaviors among Chinese patients with type 2 diabetes
Source: PLoS One. 2019 Nov 11;14(11):e0224869. doi: 10.1371/journal.pone.0224869 (PMC6844544; doi:10.1371/journal.pone.0224869)
Supplement: S3 File — (PDF) [file pone.0224869.s003.pdf]

# 山东大学医药卫生管理学院医学伦理委员会

# 审　批　件

**编号：**ECSHCMSDU20190304

项目名称：　山东省糖尿病社区防治管融合干预试点项目

项目负责人：孙强、王海鹏　　职称：教授、讲师　　联系电话：18668996362

负责研究单位：　山东大学医药卫生管理学院

合作研究单位：　山东省疾病预防控制中心

研究起止时间：　2017.10-2020.12

课题来源：山东省疾病预防控制中心（项目号：SK180022）

**审查意见：**

经本委员会审查，研究者的资格、经验符合试验要求；研究方案符合《赫尔辛基宣言》以及《涉及人的生物医学研究伦理审查办法》等国际国内法律和有关伦理规范的要求；知情同意方法适当；受试者可能遭受的风险与研究预期的受益相比适当。

同意开展该项目的研究，但在研究过程中应接受本委员会的监督，研究者在研究结束后，应当向本委员会递交最终报告，包含对于研究发现及研究结论的总结。

山东大学医药卫生管理学院伦理委员会

主任委员：[illegible]

2019年3月10日

地址：济南市文化西路44号山东大学趵突泉校区院内

联系电话：（+86 0531）88382142－8018　　联系人：朱清滋

# Ethics Committee of School of Health Care Management, Shandong University
# Approval Form

**NO:** ECSHCMSDU20190304

**Project Name:** The community-based diabetic integration of prevention, treatment and management intervention trial in Shandong province, China

**Project manager:** Qiang Sun; Haipeng Wang **Title :** Professor; Lecture **TEL:** 18668996362

**Research Institutions:** School of Health Care Management, Shandong University

**Cooperative research institution**: Shandong Provincial Center for Disease Control and Prevention

**Date: From** 2017. 10 **To** 2020.12

**Sources of Funding:** Shandong Provincial Center for Disease Control and Prevention, Grant Number: SK180022

## Ethical review:

After review by the committee, the qualifications and experience of the researchers are in line with the test requirements. The research plan is in line with the requirements of the Helsinki Declaration and the International and Domestic Laws on the Ethical Review of Human Biomedical Research and related ethical norms. The informed consent method is appropriate. The risk that the subject may be exposed is appropriate compared to the expected benefit of the study.

Thus, the committee agree to carry out the research of the project, but it should be supervised during the research process. After the study is completed, the researcher should submit a final report to the committee, including a summary of the research findings and conclusions.

School of Health Care Management, Shandong University

Signature (Chairman)：

Date:

# Translated File
